# The Recent Decision in the Vulcanite Suit

**Published:** 1874-07

**Authors:** Josiah Bacon

**Affiliations:** Treasurer.


### EDITORIAL, ETC.

---

*The Recent Decision in the Vulcanite Suit.*—BOSTON, May 8th, 1874.—*Dear Sir.*—It is my duty to notify you that the case of "Goodyear Dental Vulcanite Company vs. Dan'l H. Smith," (the case so ably contested by Henry Baldwin, Jr., Esq., and Hon. Jeremiah S. Black, the counsel employed by Sam'l S. White, of Philadelphia,) has been to day decided by the Hon. Geo. F. Shepley, Judge of the U. S. Circuit Court, in favor of the Company, upon all the points at issue. Copies of the Opinion, as delivered by the Court, may be had upon application to this office.

By request of many of the leading members of your profession, the Company has forborne to send its Agents out for collection of delinquent License fees until the decision of this case should be announced, the same seeming to be regarded as a TEST CASE upon the rights of the Company.

Now, however, this case having been fully decided, we shall require prompt payment from all parties using Rubber who have not already secured their Licenses for the present year.

We give this especial notice, as the Agents of the Company have been now sent out upon their duty of collection, and CASH PAYMENT will be required of all. You can now receive License by remitting the amount, of which you were notified, to this office, when License will be sent you by return of mail, accompanied by a copy of the opinion of the Court, in pamphlet form. More than one third of the year has now elapsed, and no note therefore, will be taken for any portion of the License fee, but CASH will be required of all without exception, and one object of this timely notice is that all may be prepared with funds on arrival of the Company's Agents, unless License shall already have been procured from this office.

Trusting, yet once again that a quiet acquiescence in our rights may cause litigation to cease, and the often asked for reduction of License fees to be made by the Company, I remain

Yours Respectfully,

JOSIAH BACON, TREASURER.

---

This notice was issued by the Rubber Company so soon after the opinion of Judge Shepley, of the U. S. Circuit Court, was delivered, that it was probably the first intimation that the majority of the practitioners of Dentistry had that this case, which was regarded with so much solicitude, had been decided adversely to their interests.

Judge Shepley says: "Upon a careful review of all the evidence in the record, I have no hesitation in coming to the conclusion that the invention of Dr. Cummings was a new and useful manufacture, that nothing appears in evidence to show that he was not the original and first inventor of the thing claimed by him, that the re-issued patent in suit is a good and valid patent, and that the defendant has infringed the same, as alleged in the bill."

This decision of a U. S. Circuit Judge has surprised many who were in hopes of being speedily relieved of what they have always regarded as an unjust tax, and to which the parties collecting it had no claim whatever. The older members of the profession, those engaged in the practice of Dentistry at the time rubber was first introduced as a base for artificial teeth, knowing that this material was so applied and used both in this country and Europe long before a patent was granted to Cummings, are not disposed to quietly acquiesce in a decision which they regard as being so unjust to themselves and others, and trust that justice may yet be meted out in that final tribunal, the Supreme Court of the U. S.

Should this last hope prove delusive, then nothing remains for those who wish to continue the use of rubber but submission, until such time as the Cummings patent shall expire, when every effort should be made to prevent its renewal.

